# RBCK1 overexpression is associated with immune cell infiltration and poor prognosis in hepatocellular carcinoma

**DOI:** 10.18632/aging.205393

**Published:** 2024-01-11

**Authors:** Jingjing Yu, Tianming Liu, Mingjiang Liu, Hu Jin, Zaiwa Wei

**Affiliations:** 1 The First Affiliated Hospital of Guangxi Medical University, Nanning, Guangxi 530021, China; 2 Department of General Surgery, The First Affiliated Hospital of Harbin Medical University, Harbin, China; 3 Department of Hepatopancreatobiliary Surgery, Yantai Yuhuangding Hospital Affiliated to Medical College of Qingdao University, Yantai, China; 4 Department of Critical Care Medicine, Liuzhou People’s Hospital, Liuzhou, China; 5 Guangxi Key Laboratory of Brain and Cognitive Neuroscience, Guilin Medical University, Guangxi, China

**Keywords:** RBCK1, immune microenvironment, liver cancer, prognosis

## Abstract

RBCK1 is an important E3 ubiquitin ligase, which plays an important role in many major diseases. However, the function and mechanism of RBCK1 in pan-cancer and its association with immune cell infiltration have not been reported. The purpose of this study is to find out the expression of RBCK1 in cancer, and to explore the relationship between RBCK1 and the prognosis of patients. Our results show that the expression of RBCK1 is up-regulated in a variety of malignant tumors, and is closely related to the prognosis of patients. Further studies have shown that RBCK1 regulates protein expression in the nucleus and plays an important role in ribosome and valine, leucine, and isoleucine degradation. Genetic variation analysis showed that RBCK1 was mainly involved in missense mutations in multiple tumors, and mutated patients showed poor prognoses. Further studies showed that RBCK1 may be interacted with proteins such as RNRPB, MCRS1, TRIB3, MKKS and ARPC3. Through protein interaction analysis, we found 43 proteins interacting with RBCK1 in liver cancer. We also analyzed immune cell infiltration and found that RBCK1 expression was positively correlated with T cells and macrophages, while it was negatively correlated with neutrophils, NK cells, and DCs in liver cancer. Finally, we confirmed experimentally that RBCK1 can significantly inhibit the apoptosis and invasion of HCC. Therefore, we speculate that RBCK1 plays an important regulatory role in the occurrence and development of HCC.

## INTRODUCTION

Liver cancer is one of the most common cancers in the world, and its incidence ranks sixth in the world [1]. More than 40% of new HCC patients are called HCC in China [2]. Hepatocellular carcinoma (HCC) is one of the most common primary liver cancer types in China, and its five-year survival rate is only 18% [3]. In-depth study of the causes, molecular mechanism and key links of the occurrence and development of HCC will help to promote the development of HCC diagnosis and treatment technology.

The RAN binding protein 2 (RanBP2)-type and C3HC4-type zinc finger containing 1 protein (RBCK1) is an E3 ubiquitin ligase protein, and its deletion can cause skeletal muscle dysfunction [4]. Accumulating studies have highlighted the potential role of RBCK1 in tumorigenesis. It has been reported that RBCK1 deletion can up-regulate the level of p53 protein and inhibit the occurrence and development in renal cell carcinoma [5]. Forced expression of RBCK1 has been documented to curb chemotherapy sensitivity in colorectal cancer [6]. RBCK1 confers different functions in different breast cancer types. Its ectopic expression augments the proliferative potential of cells by fostering the transcription of estrogen receptor alpha (ERα) and cyclin B1 in ERα-positive breast cancer cells [7]. In contrast, ectopically expressed RBCK1 restricts the progression of triple-negative breast cancer (TNBC) *in vitro* and *in vivo*, whereas RBCK1 depletion stimulates the invasive capacity of TNBC cells [8]. Although RBCK1 plays an important role in liver cancer, its role in liver cancer is not clear.

This study analyzed and validated publicly available sequencing data to elucidate the role of and the pertinent molecular mechanisms underlying RBCK1 in HCC, and provides new insights for HCC treatment and prevention.

## MATERIALS AND METHODS

### RBCK1 gene expression analysis

We study of RBCK1 mRNA expression profiles of 33 cancers by using the Tumor Immune Estimation Resource (TIMER) database (https://cistrome.shinyapps.io/timer/) [9]. To further investigate RBCK1 expression patterns, we generated box plots of differential expression using the Gene Expression Profiling Interactive Analysis (GEPIA2, version 2). which compared tumor tissues to their corresponding normal tissues from the Genotype-Tissue Expression (GTEx) database. qPCR was performed to confirm the expression of RBCK1 in HCC [10].

### Genetic cluster analysis

We used the LinkedOmics database to assess the Gene Ontology (GO) of RBCK1 [11]. The pathway enrichment analysis was performed by KEGG. The GO term analysis is divided into three categories: biological processes (BP), cellular components (CC), and molecular interactions (MF).

### Gene survival analysis and clinicopathological characteristics

The expression of RBCK1 in TCGA database and the survival rate of the corresponding patients were analyzed by R Version3.6.1. Therefore, we selected a liver cancer tissue samples that included clinicopathological data such as age, sex, grade, TNM stage, invasion depth, lymph node metastasis, distant metastasis, and vital status for further study. The measurement data of our company are based on the average and standard values. We calculated the log-rank *P*-value and hazard ratio with 95% confidence intervals. We concluded that there were statistically significant differences when *p* < 0.05. (^***^p < 0.001, ^**^p < 0.01, ^*^p < 0.05).

### Profiling of genes co-expressed with RBCK1

To explore the genetic variation and molecular mechanism of RBCK1, we screened a number of co-expressed genes with RBCK1 using the cBiofrom database (https://www.cbioportal.org/). After screening RBCK1-interacting proteins and analyzing the frequency of RBCK1 gene changes in tumors, it was discovered that RBCK1 had a high mutation rate that likely impacted cancer patient survival. Ultimately, five genes that had the strongest correlation with RBCK1 were identified.

### Establishment of protein interaction network

Searched RBCK1 interaction proteins in STRING database (https://string-db.org/) [12]. The functional proteins were studied by using protein-protein interaction network and genome-wide sequencing technology.

### Detection of immune infiltration of RBCK1 gene

The cBioPortal database and the R version 3.6.1 software were employed for studying immune infiltration in HCC microenvironment. By analyzing the correlation between RBCK1 and different immune infiltrating cells, the role of RBCK1 in the immune microenvironment was evaluated.

### Patient samples

The human liver samples of 30 cases were collected from patients at the First Affiliated Hospital of Guangxi Medical University. These patients had a confirmed diagnosis of primary hepatocellular carcinoma by clinical procedure. This experiment was approved by the Ethics Committee of Clinical Medicine at the First Hospital of Guangxi Medical University.

### Cytology and flow cytometry

Huh7 was purchased from the Cell Bank of Shanghai Institutes for Biological Sciences (Shanghai China). Cells were cultured in RPMI1640, which contains 10% fetal bovine serum (Australia, VS500T), 37°C, 98% water and 5% CO2. Use siRNA to knockdown the RBCK1 gene, according to the instructions provided by the manufacturer. The apoptosis of cells was measured by flow cytometry within 48 hours according to the manufacturer’s instructions (eBioscience, 88-8007).

### Scratch test

The ability of cell migration was evaluated by the evaluation of wound repair. Briefly, Huh7 cells were initially seeded in a confluent monolayer within 6-well plates, and then a straight scratch was made from the end of the sterile straw. The picture was taken with a microscope at either 12 to 24 hours after the injury.

### Statistical analysis

Use SPSS25 (IBM of Armonk, NY, USA) to process the data. These data are expressed as mean ± SD from at least three independent experiments. Differences between the two groups were assessed by paired or unpaired t-tests and those among multiple groups by one-way ANOVA. When *P* < 0.05, there was significant difference between the two groups.

## RESULTS

### High expression of RBCK1 gene in various cancers

**Figure 1:**
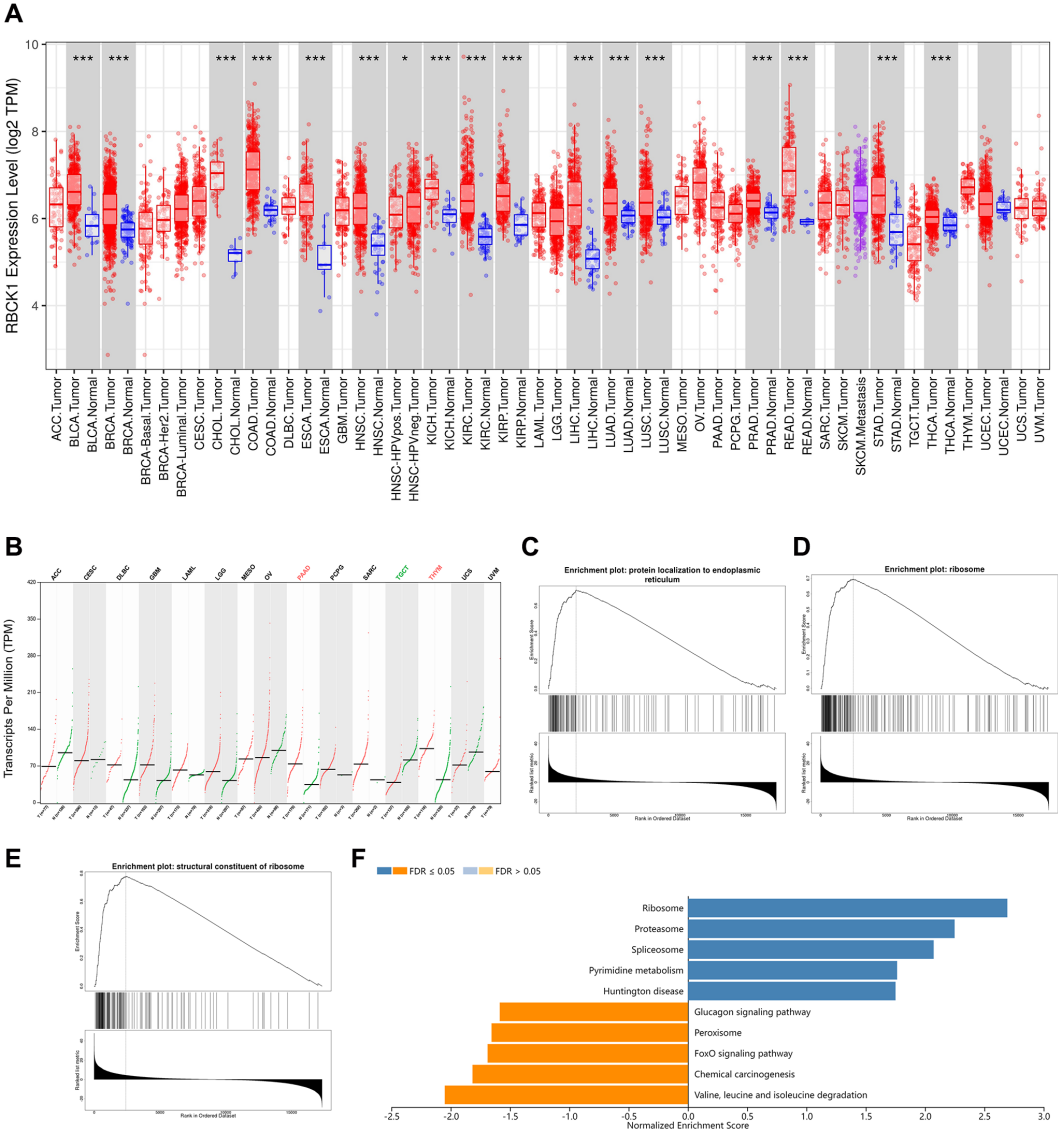
**RBCK1 is overexpression in cancers.** (**A**, **B**) RBCK1 expression in different type of cancers (TIMER2 database) and tissue (GEPIA2 database). (**C**–**E**) the partial Go terms for biological process (**C**) cellular component (**D**) and molecular function (**F**). Enrichment analysis of KEGG pathway (**E**).

We screened the expression of RBCK1 gene in a variety of cancers through TIMER2 database. It was found that RBCK1 could be detected in BLCA, BRCA, BRCA, CHOL, COAD, ESCA, HNSC, KICH, KIRC, KRIP, LIHC, LUAD, LUAD, LUSC, lung adenocarcinoma, PEAD, rectal cancer, STAD, PAAD, thymoma. However, the expression level was poor in TGCT (tracking glycoprotein cells, TGCT) tissues. For cancer types without normal controls, we use normal tissue from the GTEx data group in the GEPIA2 database as control (Figure 1A, 1B). It is speculated that RBCK1 may be an important proto-oncogene. Secondly, the GO and KEGG sequences of RBCK1 were screened out through LinkedOmics database. We found that the biological process (BP) of RBCK1 include the localization of proteins to the endoplasmic reticulum (Figure 1C), cellular components (CC) including ribosome localization (Figure 1D), and MF including ribosome composition (Figure 1E). Further KEGG pathway enrichment experiments showed that RBCK1 was positively linked with pyrimidine metabolism-related pathways while negatively linked with glucagon and fork head box O (FOXO) signaling pathways (Figure 1F).

### Relationship between RBCK1 and clinical prognosis in human tumors

**Figure 2:**
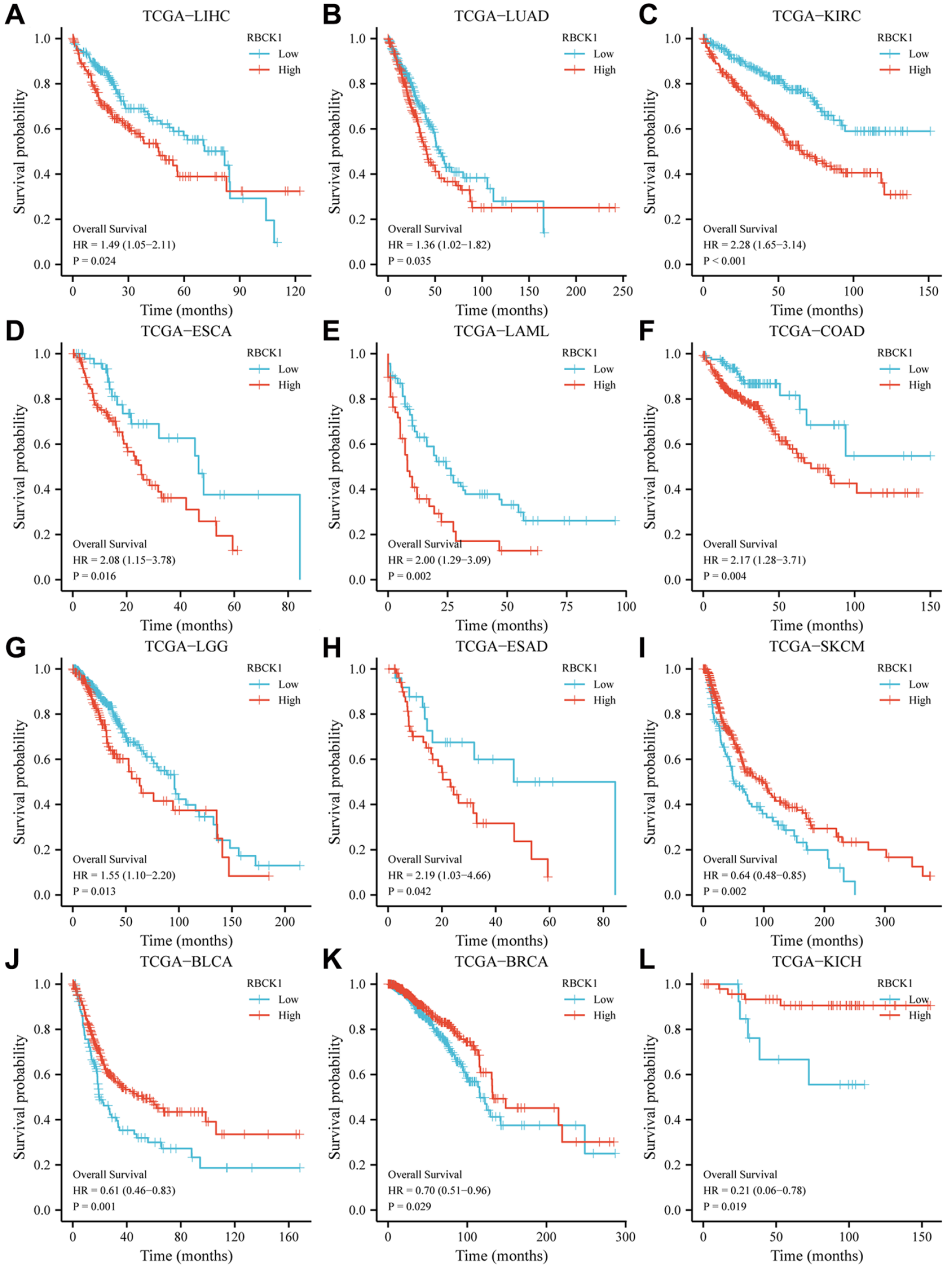
**High RBCK1 expression is generally associated with poor prognosis in various carcinomas.** Kaplan-Meier plots and statistical analysis based on the work of the TCGA Firehose Legacy study, showing the effect of increased RBCK mRNA expression within LIHC (**A**), LUAD (**B**), KIRC (**C**), ESCA (**D**), LAML (**E**), COAD (**F**), LGG (**G**), ESAD (**H**), SKCM (**I**), BLCA (**J**), BRCA (**K**) and KICH (**L**) on overall survival.

The focus of our study moved to assess the prognostic value of the differential expression of RBCK1 in several tumors. TCGA data shows that patients with high expression of RBCK1 in LIHC, LUAD, KIRC, ESCA, LAML, COAD; LGG, ESAD, SKCM and BLCA had an unfavorable prognosis (Figure 2A–2J), while that of patients with high expression of RBCK1 in BRCA and KICH had a favorable prognosis (Figure 2K, 2L). The different predictive roles of RBCK1 in different tumors may be attributed to the heterogeneity of the tumors.

### Genetic variation and co-expression analysis of RBCK1

**Figure 3:**
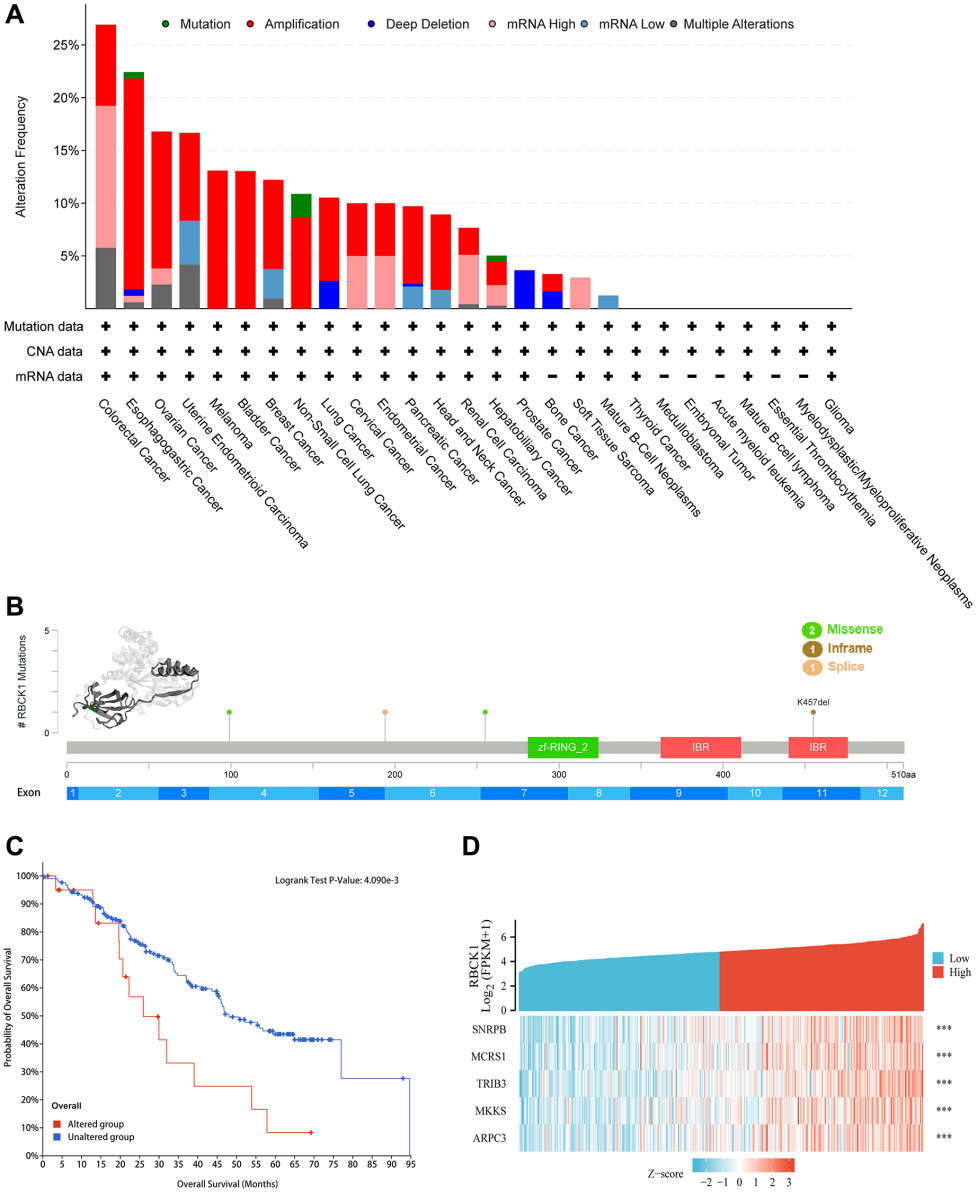
**Alteration frequency and co-expression of RBCK1 across cancers.** (**A**) alteration frequency of RBCK1 in various cancer types. (**B**) the 3D structure of RBCK1 protein and mutation sites are displayed. (**C**) Kaplan-Meier survival analysis of patients with or without RBCK1-mutant. (**D**) top 5 relative association to RBCK1.

Next, the correlation between genetic polymorphism of RBCK1 and cancer was studied by using cBioPortal database. The study found that RBCK1 mutated in all 19 cancers, with the frequency of variation exceeding 5% in 15 tumor types, 10% in 11, 15% in 4, 20% in 2, and >25% in COAD (Figure 3A). As can be seen from Figure 3B, the 3D structure of RBCK1 shows the locus and the specific genetic changes dominated (Figure 3B). Secondly, combined with TCGA data, we assessed the effects of RBCK1 genetic variants on patient survival and prognosis. Results show that RBCK1 gene mutation is closely related to tumor prognosis (*P <* 0.05) (Figure 3C). In addition, we also screen the co-expressed genes with RBCK1 and five gene including small nuclear RNA binding proteins (snRBPs), microspherule protein 1 (MCRS1), Tribbles homolog 3 (TRIB3), *M*cKusick–Kaufman/Bardet–Biedl syndromes putative chaperonin (MKKS), and actin-related protein 2/3 complex subunit 3 (ARPC3) were be found (Figure 3D).

### Study on hepatocyte-specific PPI network of RBCK1

**Figure 4:**
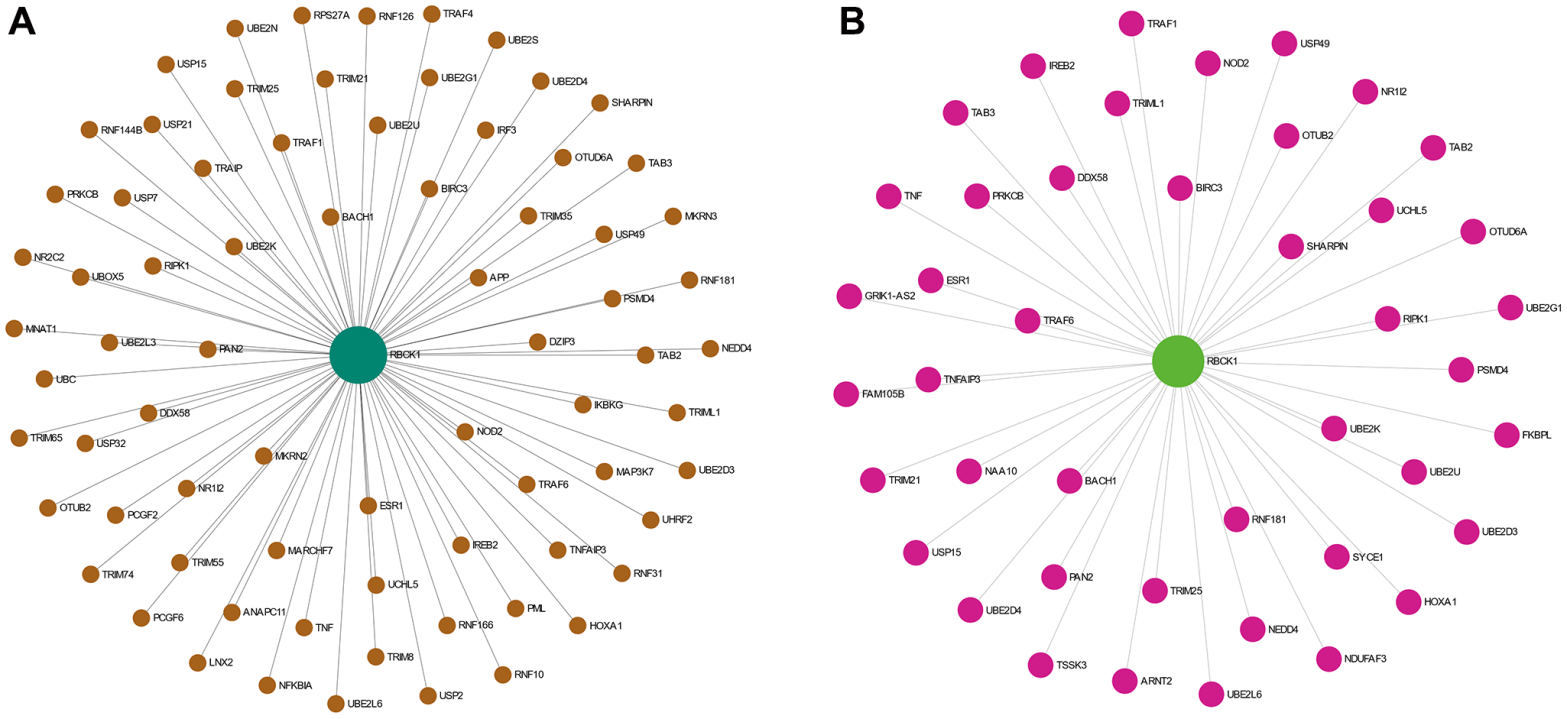
**The PPI network analysis of RBCK1.** (**A**) PPI network analysis of RBCK1 in the co-expression network. (**B**) the liver-specific protein-protein interaction network of significantly RBCK1 co-occurrence genes.

The proteins interacting with RBCK1 were screened by STRING database. we found that RBCK1 could bind to 65 proteins (Figure 4A), while 43 proteins were found to bind to RBCK1 protein in hepatocellular carcinoma (Figure 4B).

### Immune infiltration analysis of RBCK1 in HCC and its clinical relevance

**Figure 5:**
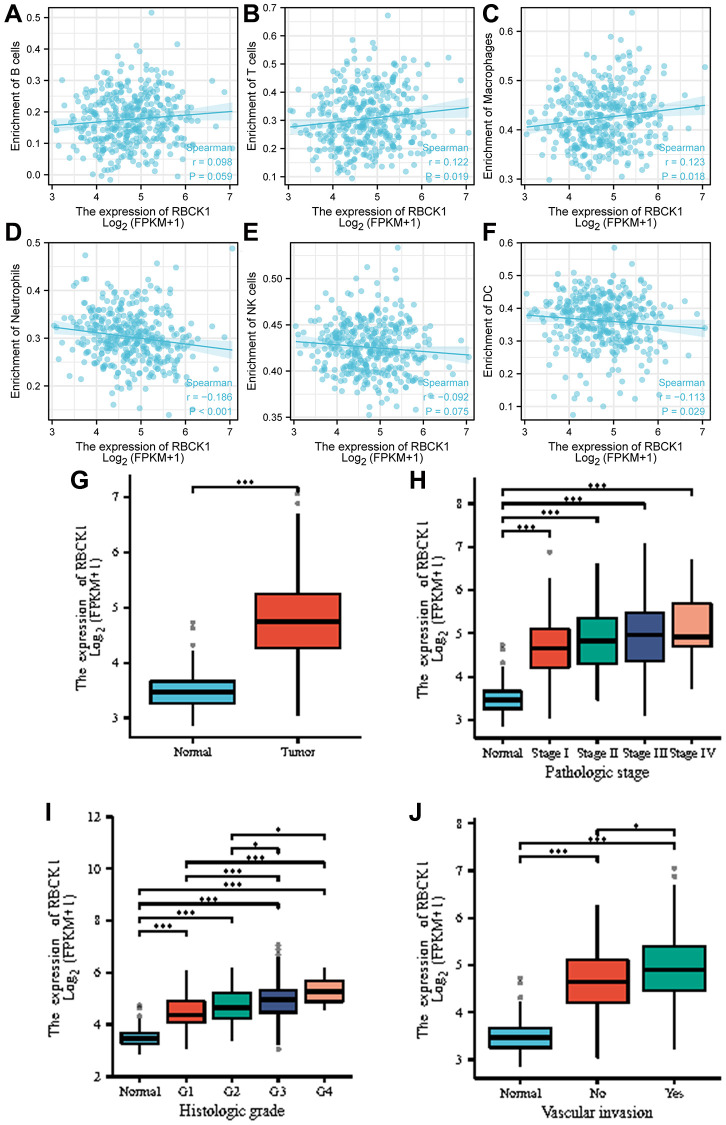
**Correlation analysis between RBCK1 levels and clinicopathological characteristics of HCC patients.** (**A**–**F**), relationship between RBCK1 and B cells (**A**) T cells (**B**) Macrophages (**C**) Neutrophils (**D**) NK cells (**E**) and DC (**F**). (**G**) RBCK1 is overexpressed in HCC patients. (**H**–**J**) clinical analysis the relationship between RBCK1 and histologic grade (**H**) pathologic stage (**I**) and vascular invasion (**J**) of HCC.

However, the role and molecular mechanism of RBCK1 in the occurrence and development of hepatocellular carcinoma are still unclear. Our data suggests that RBCK1 is closely associated with the immune microenvironment, with its expression being positively correlated with B cells, T cells, and macrophages, while demonstrating a negative correlation with neutrophils, NK cells, and DCs (Figure 5A–5F). Furthermore, data also reveal that RBCK1 was highly expressed in HCC (Figure 5G), and was closely related to pathological grade and microvascular infiltration (Figure 5H–5J).

### RBCK1 drives proliferation and inhibits apoptosis of HCC cells

**Figure 6:**
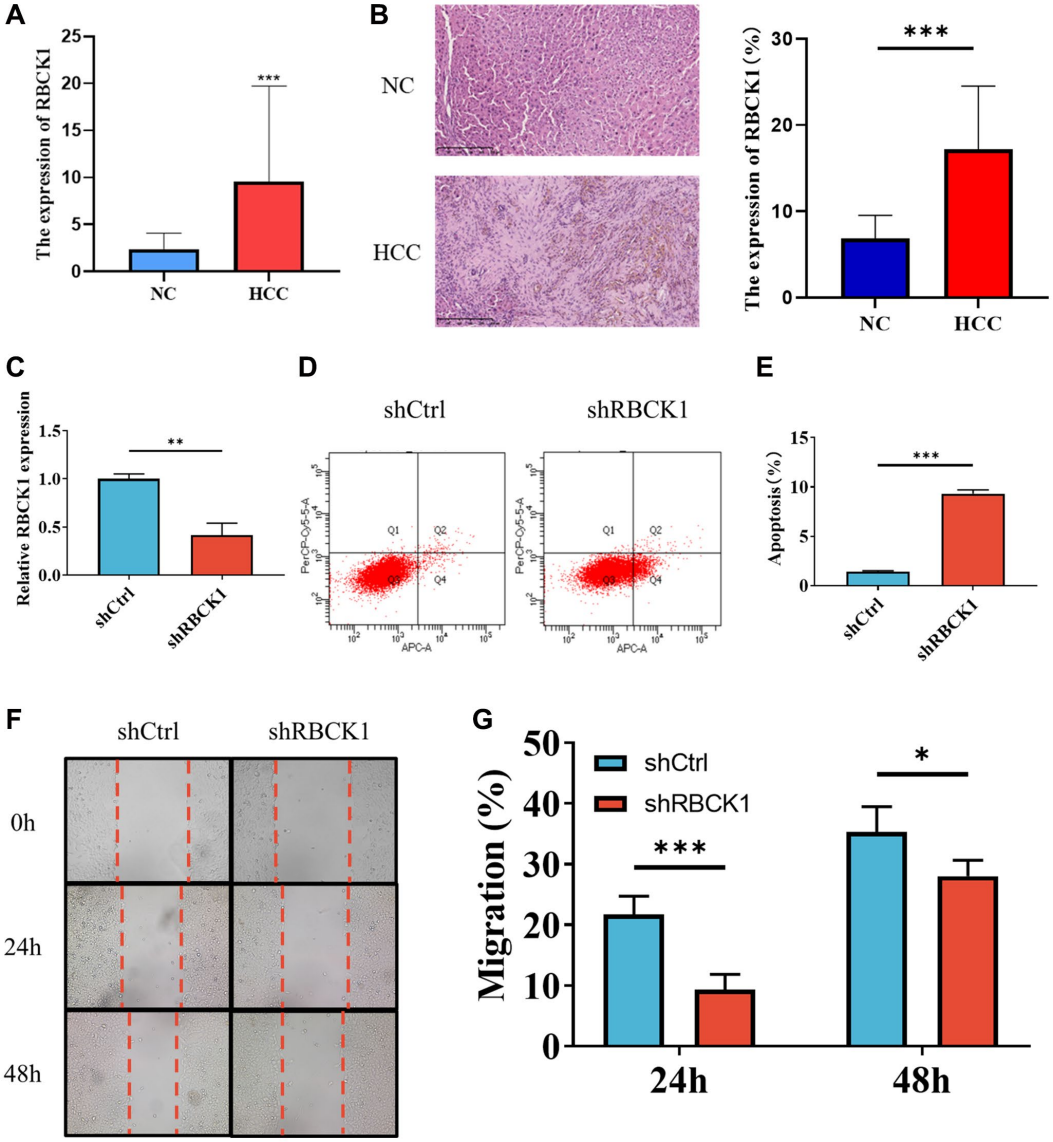
**Knockdown of RBCK1 inhibits cell proliferation and promotes apoptosis in HCC cell lines.** (**A**, **B**) RBCK1 expression in 30 cases of HCC and its adjacent tissues from the First Affiliated Hospital of Guangxi Medical University by qRT-PCR (**A**) and IHC (**B**). (**C**) The knockdown efficiency of the RBCK1 gene. (**D**, **E**) The level of apoptosis in Huh7 cells after RBCK1 gene knockdown. (**F**, **G**) The cell scratch assay was performed to evaluate the migration ability of Huh7 cells after RBCK1 gene knockdown.

The relationship between RBCK1 and HCC is further clarified in HCC and paracancerous tissues. The expression of RBCK1 mRNA in HCC was higher than that in paracancerous. (Figure 6A). Immunohistochemical results suggesting that RBCK1 is located in the cytoplasm (Figure 6B). The highest knockdown efficiency for the RBCK1 gene was observed with shRBCK1. (Figure 6C) Flow cytometric detection found that RBCK1 can induce apoptosis in Huh7 cells, suggesting that RBCK1 may be the key molecule to regulate the apoptosis of Huh7 cells (Figure 6D, 6E). Further study found that RBCK1 deletion can significantly affect the migration of Huh7 cells (Figure 6F, 6G). Take these together, our results show that RBCK1 play a cancer-promoting role in HCC.

## DISCUSSION

Previous studies had showed RBCK1 was originally identified as an important component of the linear ubiquitin assembly complex, stimulating nuclear factor-kappa B (NF-κB) signaling activation in the immune response and playing different roles in tumorigenesis [8]. As described by Chen et al., RBCK1 is highly expressed in HCC and stabilizes ring finger protein 31 (RNF31) by expediting its metastatic potential and growth [13]. Additionally, Xu et al. found that RBCK1 appreciably facilitated the invasive and metastatic capacity of HCC by enhancing glucose transporter 1 (GLUT1)-dependent aerobic glycolysis and disrupting the PPARγ or PPARγ/PGC1α complex [14].

In this study, we verified that RBCK1 precipitated migration and curbed HCC apoptosis by using bioinformatics prediction and experimental validation, and the exploration of genetic variation and immune cell infiltration also provided novel mechanistic insights. The obtained data suggested that RBCK1 was expressed at higher levels in a wide array of tumors relative to normal tissues, especially digestive system tumors such as CHOL, COAD, LIHC, READ, STAD, and PAAD. This suggests that RBCK1 might be a proto-oncogene in the digestive system. Single gene GO and KEGG enrichment analyses further showed that RBCK1 was localized in the ribosome and involved in the ribosomal structural composition. It is metabolically related as a ubiquitinating enzyme and negatively associated with the FOXO signaling pathway. Interestingly, concurring with our findings, Lei et al. have speculated that the transcription factor FoxO1 is a negative regulator of virus-triggered interferon-β induction during viral infection and mediates IFN regulatory factor 3 (IRF3) degradation independent of that by RBCK1, the common E3 ubiquitin ligase [15].

After determining RBCK1 overexpression in various tumors, except BRCA and KICH, is positively correlated with poor prognosis, we analyzed the relationship between genetic variants of RBCK1 and tumors. RBCK1 genetic variation occurred in multiple tumors and was detrimental to patient survival. Among the five genes with the strongest correlation with RBCK1 expression, MCRS1 was a novel target of microRNA (miR)-186 in HCC cells [16], and MCRS1 expression abrogated the inhibitory effects of miR-186 on the metastatic capacity and Wnt/β-catenin signaling contributing to HCC cell migration. Moreover, TRIB3 favors HCC cell growth and cell cycle entry through activation of the mitogen-activated protein kinase (MAPK) pathway [17]. The involvement of ARPC3 as a possible key gene has also been documented in the carcinogenesis of hepatitis delta virus (HDV)-HCC, offering potential as a prognostic indicator [18]. Hence, we speculated that RBCK1 might be implicated in the mechanism of hepatocarcinogenesis and enhance its metastasis and growth.

Further investigation suggested that among the RBCK1-interacting proteins, both generally and in the liver, BACH1, RIPK1, UBE2K, and BIRC3, all potently interacted with RBCK1 and were associated with the malignant progression of HCC. BACH1 is a transcription factor implicated in modulating oxidative stress [19], cell cycle progression [20], heme homeostasis [21], inflammation, and immunity. Ectopic expression of BACH1 augments malignancy and metastatic capacity of HCC by inducing expression of cell motility-related genes IGF1R and PTK2 [22]. The necroptosis-driving gene RIPK1 [23] blunts the TRAF2-dependent pathway in HCC [24], whereas repression of RIPK1 kinase activity-mediated apoptotic potential prevents steatohepatitis and HCC [25]. UBE2K, similar to RBCK1, acts as a ubiquitin ligase and interacts with c-Myc to play an oncogenic role in HCC progression through the UBE2K/c-Myc axis [26]. Notably, cell apoptosis could be curtailed by BIRC3 via binding to TRAF1 and TRAF2 [27]. BIRC3 up-regulates MAP3K7 levels to stimulate ERK1/2 phosphorylation, thereby driving epithelial-mesenchymal transition and the migratory and metastatic capacity of HCC *in vitro* and *in vivo* [28, 29]. Thus, proteins strongly interacting with RBCK1 in the liver can be mostly considered biomarkers of HCC, suggesting the potential role of RBCK1 in accelerating HCC malignancy.

Based on the differential expression of RBCK1, we assessed the effect of RBCK1 in different tumor-infiltrating immune cells in HCC. Significant expression of RBCK1 was noted in five subtypes of immune cells: T cells, macrophages, neutrophils, NK cells, and DCs. A positive but not significant correlation was identified in B cells. There was a significant difference in the ratio of immune cells according to the expression of RBCK1. These findings implied that RBCK1 levels were tightly associated with tumor-infiltrating immune cells. RBCK1 and its co-expressed genes may be involved in the immune response to hepatocarcinogenesis, leading to unfavorable prognosis in HCC patients. We speculate that high RBCK1 expression might affect macrophage activities [30] and drive the polarization of macrophages to M2 phenotype and eventual differentiation into tumor-associated macrophages (TAMs), which are critical players in the invasive, metastatic, and angiogenic potential of tumors [31, 32]. RBCK1 expression was positively associated with microvascular invasion, as evidenced by the results of clinical staging and grading and microvascular invasion analyses in patients with HCC.

Finally, clinical samples of HCC and paracancerous tissues were harvested to further validate RBCK1 expression in HCC and its ability to influence hepatocarcinogenesis. RBCK1 was observed to be localized in the cytoplasm and highly expressed in HCC tissues, which was consistent with the prediction by bioinformatics analysis. Furthermore, RBCK1 knockdown caused a significant increase in the apoptotic ability of HCC cells. This concurs with the findings of Ikeda et al. showing that RBCK1 downregulation caused by SHARPIN deficiency further enhanced apoptotic events [33]. Consistently, RBCK1 downregulation induced apoptosis and curtailed proliferative and migratory phenotypes in ovarian cancer cells [34]. Therefore, we suggest that RBCK1 might favor the BP of HCC by retarding apoptosis and driving the migratory events.

## CONCLUSION

RBCK1, an E3 ubiquitin ligase, is highly expressed in HCC. Its expression, interacting proteins, and function in the prognosis of HCC were examined by bioinformatics analyses combined with experimental support. We found that RBCK1 facilitated HCC progression by affecting microvascular invasion, cell migration, and apoptosis, revealing its potential role in tumor immunology and promising value as a prognostic biomarker and therapeutic target for HCC.
